# EPS—Then and Now

**DOI:** 10.3390/microorganisms4040041

**Published:** 2016-11-18

**Authors:** Hans-Curt Flemming

**Affiliations:** 1 Biofilm Centre, University of Duisburg-Essen, Universitätsstrasse 5, Essen 45141, Germany; hc.flemming@uni-due.de; 2 Singapore Center for Life Science Engineering (SCELSE), Nanyang Technological University, Singapore 637551, Singapore; 3 Water Academy, Licherstrasse 24, Frankfurt 60389, Germany

**Keywords:** biofilms, EPS, emergent properties, nutrient acquisition, tolerance, resistance

## Abstract

“Slime” played a brief and spectacular role in the 19th century founded by the theory of primordial slime by Ernst Haeckel. However, that substance was never found and eventually abandoned. Further scientific attention slowly began in the 1930s referring to slime as a microbial product and then was inspired by “How bacteria stick” by Costerton et al. in 1978, and the matrix material was considered to be polysaccharides. Later, it turned out that proteins, nucleic acids and lipids were major other constituents of the extracellular polymeric substances (EPS), an acronym which was highly discussed. The role of the EPS matrix turns out to be fundamental for biofilms, in terms of keeping cells in proximity and allowing for extended interaction, resource capture, mechanical strength and other properties, which emerge from the life of biofilm organisms, including enhanced tolerance to antimicrobials and other stress. The EPS components are extremely complex and dynamic and fulfil many functional roles, turning biofilms into the most ubiquitous and successful form of life on Earth.

## 1. Too Good to Be True?

**Figure 1:**
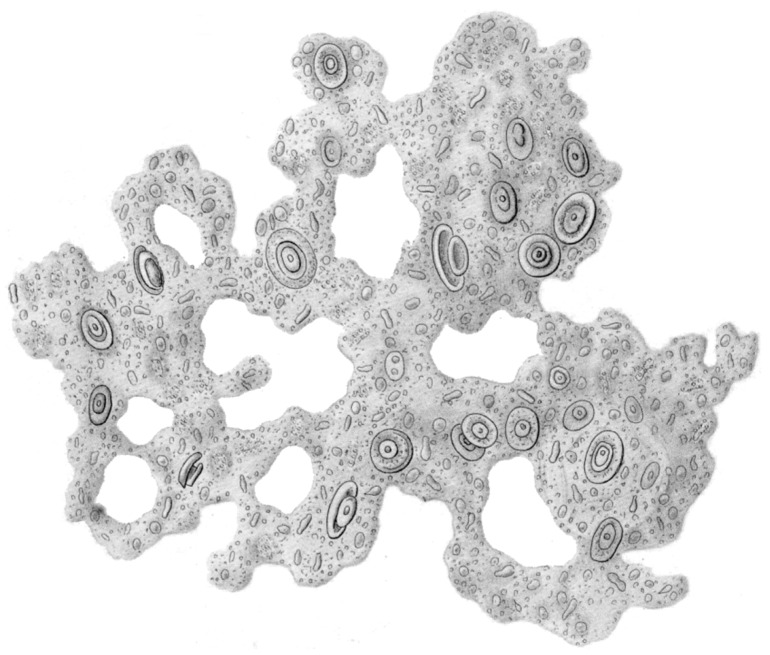
*Bathybius Haeckelii*. Figure 1 on Table 17 in Ernst Haeckels Article, *Beiträge zur Plastidentheorie* (1870) (Image in public domain).

It was such a great idea! In “The History of Creation”, the German biologist Ernst Haeckel hypothesized in 1868 that life originated from primordial slime [1]. He postulated that this substance was constantly coming into being at the bottom of the ocean. Moreover, Thomas Henry Huxley, a British biologist, studied mud from the Atlantic seafloor, discovering an albumineous slime which appeared to him to be exactly that primordial slime. To Haeckel’s honour, he termed it *Bathybius haeckelii* and was convinced to have found the missing link between matter and organic life (Figure 1).

Huxley speculated that *Bathybius* formed a continuous mat of living protoplasm that covered the entire ocean floor, in perfect accordance with Haeckel’s theory. Unfortunately, the Challenger expedition from 1875, exploring the sea floor, found none of it so far, and later it turned out that *Bathybius* only was a precipitate of calcium sulphate precipitated from seawater by the alcohol used to preserve Huxley’s samples [2]). Rice [3] assumes, however, it was the crude sea floor sampling technique of the Challenger scientists stirring up the sedimented marine snow, which would have come much closer to the appearance of the postulated “primordial slime”. That was a near miss of a paramount role of slime in the history of life as the bridge between non-living and living matter and the key to “abiogenesis”: the evolution of living organisms from abiotic matter! It sank into oblivion, but later, it was revealed that its role was fundamental in a slightly different sense, as will be outlined in this overview.

## 2. Slime

For quite some decades after, slime was no object of research. The next serious record was the publication of Beckwith from 1931 [4], describing slime as a nuisance in paper production. He analysed the microbial flora, confirmed the microbial origin of slime and found it mainly consisting of polysaccharide. “The carbohydrate content of the white water (white water is the suspension of cellulose fibres and many additives for paper production) due to hydrolysis of cellulose stimulates formation of bacterial gum. With formation of this mucilaginous coating, floating bits of cellulose are caught together with other finely granular detritus. This enmeshed mass contains the many bacterial organisms which constitute in part the flora of white water”. That description is still accurate today. He attributed slime formation to capsule-forming bacteria, which could not be confirmed—it is a plethora of bacteria forming slime in paper mills [5].

Further progress was achieved by the use of submerged slides to investigate microbial adhesion, a common technique already in the 1930s (e.g., Cholodny [6]). In thorough investigations on the attachment of bacteria to glass surfaces, in 1943, ZoBell was trying to find out “how bacteria attach”. He observed that “Most sessile bacteria are found with the bacterial cells in intimate contact with the solid surface. It is believed that after coming into contact with a solid surface, physiologically active sessile bacteria secrete a cementing substance. When the bacteria are removed mechanically from glass slides to which they have attached themselves, a faintly staining film having the shape and arrangement pattern of the attached cells frequently remains on the slides” [7]. Such “footprints” have been confirmed in detail a few decades later by Neu and Marshall in 1991 [8]. ZoBell also assumed that extracellular enzymes are retained in the film and contribute to nutrient acquisition of film organisms [9,10]. Heukelekian and Heller [11] saw more biological advantages of the film: “Surfaces enable bacteria to develop in substrates otherwise too dilute for growth. Development takes place either as a bacterial slime or colonial growth attached to the surfaces. Once a biologically active slime is established on surfaces, the rate of biological reaction is greatly accelerated” [11].

Zobell and Allen [10] reported in 1935 that the film appeared to be a part of or product of the bacterial cell. The fact that it was most pronounced in dilute nutrient solutions made it seem probable that the film did not simply consist of organic matter adsorbed from the water. “However, it is still indeterminate whether the bacteria are responsible for the presence of the film or if the film of adsorbed organic matter precedes the bacteria thereby providing for their attachment. Microscopic particles of detritus including particles of carbon and indigotin, with which the slides had been treated, stick to the film thereby establishing that the film has adhesive properties. This mucilaginous slime produced by sessile bacteria is believed to be instrumental in the fouling of submerged surfaces” [10].

A wake-up call was the seminal publication 1978 in Scientific American: “*How bacteria stick*”, by Bill Costerton, Gill Geesey and K.-J. Cheng [12]. It drew the attention to the matrix, which was seen to keep bacteria together and to attach them to surfaces. The authors stressed the difference between laboratory cultures and the life of microorganisms in the natural environment. Key point was that only the latter ones were shown to be embedded in a mesh of fibers, called “glycocalyx” in analogy to the surface of eukaryotic cells. Based on scanning electron microscopy evidence, they considered the matrix which embeds biofilm bacteria mainly as consisting of “polysaccharide fibres, fabricated and oriented by the cell itself”. They found that a bacterium can anchor itself to a substrate “by spinning a mat of polysaccharide fibers that withstands enormous shear forces… on the surface of sedimentary rocks in mountain streams, the “glycocalyx” prevents washing out of the attached bacteria” and provides a conveyor band-similar situation “in which the water flow supplies nutrients to the sedimentary organisms even at low concentrations”. They too confirmed the role of the matrix for nutrient acquisition: “The fibers of the glycocalyx may not only position bacteria but also conserve and concentrate the digestive enzymes released by the bacteria”. In fact, the matrix represents an activated reaction space, ideally suited for resource capture. This is enabled by the sorptive properties of biofilms as well as by the retention and accumulation of extracellular enzymes [13], resulting in an acquired nutrient reservoir with a digestion system external to the cells due to interaction between enzymes and polysaccharides. In addition to degrading sorbed substances, the extracellular enzymes can also attack the substratum, leading both to biodegradation of particulate matter and, thus, contribute to the global self-purification processes on Earth. Such processes, however, can occur in the wrong place and at the wrong time from a human perspective and known as biodeterioration [14]. The matrix represents an ultimate recycling yard, which contributes to optimal resource capture and exploitation, and it represents a huge fraction of the reduced-carbon reservoir in soils, sediments, and suspended aggregates such as marine, lake and riverine snow, providing nutrients and, thus, enhancing the survival of microorganisms [15,16].

**Figure 2:**
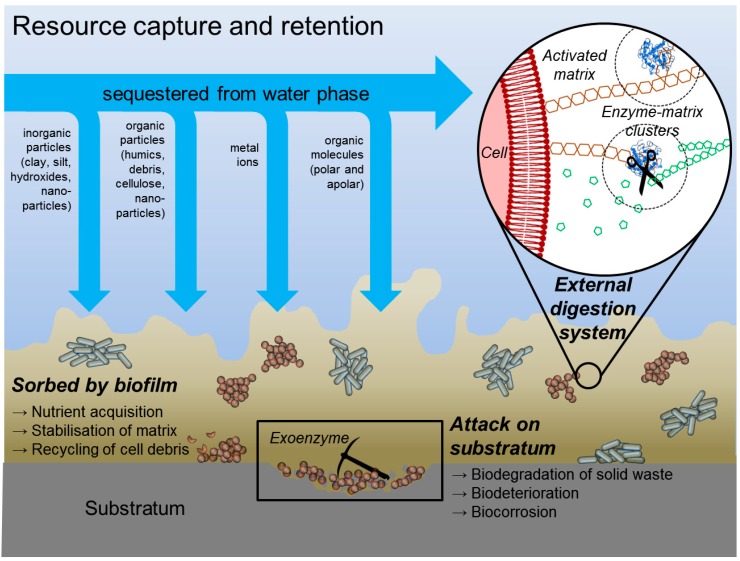
Resource caption and retention by and in biofilms. The biofilm is a sponge-like system that provides surfaces for the sorption of a diverse range of molecules that can be sequestered from the environment. This confers several benefits to the biofilm, such as nutrient acquisition and matrix stabilization. Similarly, the physicochemical properties of the matrix enable biofilms to retain and stabilize extracellular digestive enzymes produced by biofilm cells, turning the matrix into an external digestive system. Surface-attached biofilms are not only able to take up nutrients from the water phase but can also digest biodegradable components from the substratum, which is exposed to enzymes in the matrix (after [19], with permission).

Geesey suggested [17,18] that “the close physical relations are believed to promote efficient transfer of nutrients from one organism to the other”. This was another hint to the concept of the matrix as an external digestion system, an idea that has later been expanded and elaborated as a concept of resource capture by biofilm organisms and their matrix (Figure 2 after [19], with permission).

An example for the retention of exoenzymes and thus, the enzymatic activation of the matrix, is the interaction between lipase and alginate in *P. aeruginosa* as confirmed by biochemical experiments and molecular modelling [20]. Positively-charged amino acids are located opposite to the active centre and interact with the negatively-charged groups of the alginate moiety.

Visionary, Costerton and his colleagues suggested, “*that antibiotics may not be able to overcome the binding capacity of the glycocalyx in to reach their bacterial target*” [12]. Because of the fundamentally important aspects of this observation, it has increasingly become the object of intense scientific research (e.g., [21,22]. However, the mechanisms for the enhanced tolerance and resistance of biofilm organisms to antibiotics, disinfectants and other stress are still not fully understood.

## 3. The Term EPS

The term “glycocalyx” was soon abandoned in favour to “extracellular polymeric substances” (EPS), e.g., by Geesey [18]: “These substances, which, for the most part, are of biological origin, participate in the formation of microbial aggregates… In nature, EPS exist as an extremely hydrated (98% water) gel consisting of a network of fibers elaborated by and encapsulating a consortium of microbial species, which may include both prokaryotic and eukaryotic cells”. At that point, he still considered the EPS matrix as mainly fibrillar, based on electron microscopy. However, he added a caveat concerning the effect of dehydration prior to microscopy, leaving only ca. 2% of solid matter which is then seen in scanning electron micrographs. This generates strong artifacts of an otherwise highly hydrated structure. The fibres were considered mainly polysaccharides, but the presence of proteins was also acknowledged.

In the glossary to the report of the Dahlem Workshop on Structure and Function of Biofilms 1988 in Berlin [23], EPS were defined as “*organic polymers of microbial origin which in biofilm systems are frequently responsible for binding cells and other particulate materials together (cohesion) and to the substratum (adhesion)*”.

EPS slowly gained the attention of more researchers, acknowledging the fundamental role of the matrix. One result was a book published in 1999 by Wingender et al. [24]. Here, the role of proteins as an important, constituent and significant proportion of EPS was pointed out. The term had been expanded from “exopolysaccharides” to the more general form as “extracellular polymeric substances”. In that book, it was strongly stressed that the isolation procedure is crucial for the obtained classes and amounts of EPS [25]—in other words: different isolation methods lead to different EPS components.

In 1992, Luis Melo, by that time at the University of Braga in Portugal, hosted an international workshop on biofilms in the wonderful Algarve. He could attract early biofilm protagonists such as Bill Characklis, James Bryers, Madilyn Fletcher, Henk Busscher, Jean-Claude Block, Thomas Neu and others, and I had the fortunate opportunity to attend. In addition, it was there, when we informally decided in our own right to define EPS as “extracellular polymeric substances”, which included proteins, nucleic acids and lipids, as opposed to “exopolysacccharides” which would cover only one component of the matrix. Afterwards, we went to the disco and danced, very happy with ourselves, but unaware of the heated discussion which raged about shifting the meaning of the acronym EPS from exopolysaccharides to extracellular polymeric substances. This was sorted out in the legendary Biofilm Club in the remote country mansion of Gregynogh. The result was a very profound, important and straightforward chapter in the book “Biofilms: Order from chaos?” with the title “EPS: what’s in an acronym?” [26]. Here, Ian Sutherland, the outstanding pioneer of bacterial exopolysaccharides [27,28] firmly stated that the acronym EPS had a sound history (with one of the first citations by Tsien and Schmidt in 1977 [29]): “In almost 2000 papers in the biochemical literature the term ‘exopolysaccharides’ can be found and is very frequently abbreviated to EPS (over 1600 of these)” and he found it very unfortunate that the terms had been confused. However, it was too late: “… the term EPS for extracellular polymeric substances evolved, together with the research on complex, multi-species biofilms. In this respect, the term EPS… has become a standard term of scientist in the biofilm research field”, referring to Wingender et al. [24,30]. Further down, it is stated “Everybody knows what is meant, even if many scientists still neglect the huge variety of possible polymers and usually think about polysaccharides only… the term EPS is now used… for matrix polymers which include polysaccharides, proteins, nucleic acids and amphiphilic polymers.… All these polymers together form the complex matrix in extended microbial communities that may be found in the form of mobile aggregates, stationary films and mats”. Opening the window to further relevance of EPS the authors stated “To add to the complexity, not only are there a variety of different extracellular polymers present in the matrix, but also many of these polymers are likely to interact with one another depending upon their chemical nature and locality” [26]. At the end of their chapter, the authors suggested to use the term “EXPS” for Extracellular polymeric substance”, but that did not gravitate into scientific literature. Since then, the discussion about the acronym has been practically terminated with “extracellular polymeric substances” as the meaning of EPS.

## 4. The Structure of EPS

**Figure 3:**
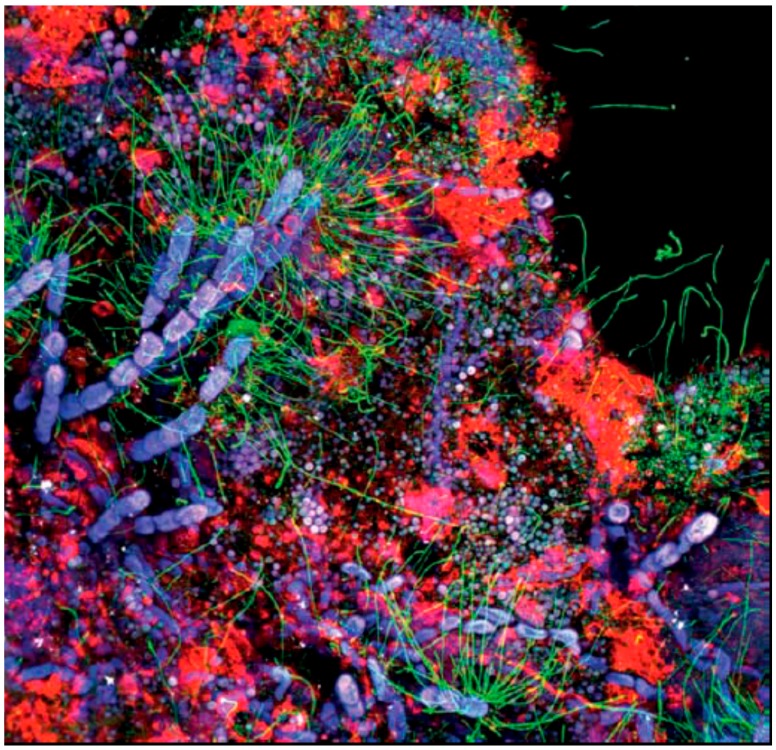
Fluorescent staining of a biofilm. Colour allocation: Nucleic acid stain (SybrGreen) = green; lectin stain (AAL-Alexa568) = red; autofluorescence of algae (chlorophyll A) = blue; autofluoresence of cyanobacteria = purple/white. Image dimensions: 246 × 246 μm (from [34], with permission, source: [35]).

Already in 1978, Geesey and coworkers predicted that biofilms are not just a piling up of bacteria but well structured. However, it was the work of Thomas Neu and John Lawrence [31,32,33], using confocal laser scanning microscopy (CLSM) and generated fantastic images of this complexity, clearly revealing that the EPS matrix is highly structured with distinct components and zones, and not just an amorphous gel. They developed the analytical use of fluorescent staining of the biofilm matrix not only into a very important scientific tool but also showed the vast variability of biofilm components and produced veritable pieces of art. A striking example is Figure 3, the cover picture of the second book on EPS [34]. It reveals the incredibly complex structure of environmental biofilms. This example shows maximum intensity projection of a river biofilm developed on a pebble stone. The biofilm with a thickness of 186 μm was examined for its autofluorescence and stained for nucleic acids as well as for glycoconjugates. Take notice of one class of biofilm matrix glycoconjugates shown in red. In addition, a symbiotic interaction between algal filaments (blue) and filamentous bacteria (green) can be seen.

An example for the complex interactions between matrix components has been demonstrated in detail in *Escherichia coli* K-12. Here, Serra et al. [36] report “using scanning electron and fluorescence microscopy, cellulose filaments, sheets and nanocomposites with curli fibers were localized in situ at cellular resolution within the physiologically two-layered macrocolony biofilms of this “de-domesticated” strain. As an architectural element, cellulose confers cohesion and elasticity, i.e., tissue-like properties which—together with the cell-encasing curli fiber network and geometrical constraints in a growing colony—explain the formation of long and high ridges and elaborate wrinkles of wild-type macrocolonies. In contrast, a biofilm matrix consisting of the curli fiber network only is brittle and breaks into a pattern of concentric dome-shaped rings separated by deep crevices”.

Another milestone in exploration of EPS matrix fine structure was the introduction of microelectrodes into biofilm research in 1988 by Revsbech, Jørgensen, and de Beer [37,38]. Oxygen distribution could be visualized in micrometers of local resolution, showing how anaerobic organisms could live well in aerobic environments while aerobes were consuming it faster than it could diffuse, generating anaerobic pockets [39].

## 5. Functions of EPS

It was a long way from EPS as an amorphous matrix to the differentiated functional view as shown in Figure 1 and Figure 2. This view had changed dramatically over the years. Biofilms were likened to “cities of microbes” [40] with the EPS matrix as the “house of the biofilm cells” [41]. Such anthropocentric analogies reflect complex social interactions, based on life in the matrix. In fact, the matrix was identified as the stage for the emergence of the remarkable properties of biofilms. In a recent publication, the success of the biofilm mode of life has been associated with the emergent properties, which arise from aggregated cells, kept together by the biofilm matrix [19]. Emergent properties are novel structures, activities, patterns and properties, which arise during the process, and as a consequence, of self-organization in complex systems [42]. For microbiological communities, emergent properties of a community are “characteristics not identifiable by analyzing the component organisms in isolation” [43].

**Figure 4:**
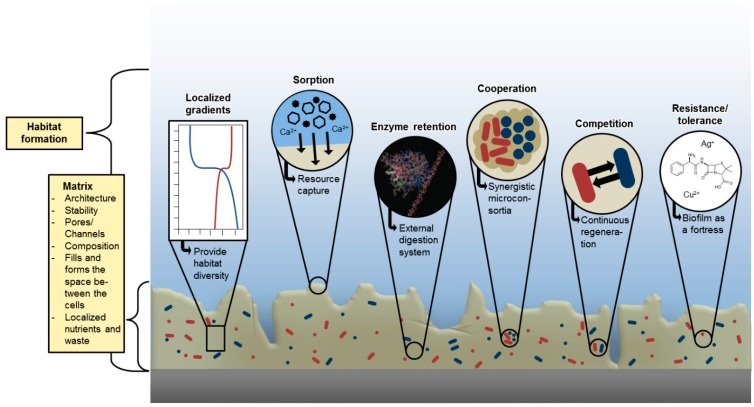
Properties of biofilms emerging from life in the EPS matrix (after [19], with permission).

A consequence is the biogenic formation of the biofilm habitat, characterized by strong gradients, high biodiversity, and complex, dynamic and synergistic interactions including enhanced horizontal gene transfer and true features of multicellularity. The primary emergent property of biofilms is based on the fact that all of these characteristics are present concomitantly with the matrix as scaffold (Figure 4 after [19], with permission).

The components agglomerated in the matrix are not just a heap of macromolecules but fulfil many important functions in the life of biofilm cells [44]. The functions of EPS components first were compiled in a review by Thomas Neu and John Lawrence [45] and have been differentiated since [19], as shown in Table 1.

After excretion by the cells, the EPS can be modified along the biofilm life cycle — the biofilm cells continuously remodel their immediate environment. Some of the possible modifications are listed in Table 2 [48].

It is the EPS matrix that makes high cell densities, the proximity of the cells in long-term arrangements, and the internal structure of biofilms possible [50]. Densities of cells within biofilms are surprisingly rarely reported, but where they appear to be significantly higher than for planktonic cells (Table 3).

## 6. Extracellular DNA

Extracellular nucleic acids deserve specific attention as they have been the most overlooked and underestimated component of the EPS. However, they turn out to be of surprising and complex relevance. Nucleic acids have been found in the EPS matrix for very long time, but usually interpreted as the leftovers from lysed cells (see Catlin, 1956 [57]) without further attention. However, it was Whitchurch et al. in 2001 [58] who reported that, surprisingly, extracellular DNA (eDNA) was required for the formation of *Pseudomonas aeruginosa* biofilms and, thus, had a function. Villain et al. [59] demonstrated, that eDNA is important for adhesion during attachment of *Bacillus cereus* cells and for different species eDNA was essential during the early stages in biofilm formation [59,60]. In addition, a structural function has been suggested, for instance for *Staphylococcus aureus* where it is linking beta toxin in the presence of eDNA, resulting in skeletal framework as basis for biofilm formation [61]. For *P. putida* the presence of the TOL-plasmid (responsible for toluene and xylene mineralization) increases the amount of eDNA and results in the formation of pellicles and thick biofilms [62]. These effects are likely to be mediated by the physical properties of eDNA, which increases the cell surface hydrophobicity and this also increases adhesion to hydrophobic surfaces for *Streptococcus mutans* [63]. Interestingly, in *P. aeruginosa,* eDNA interacts with Psl and contributes significantly to the mechanical stability of the EPS matrix [64]. Furthermore, it was found that eDNA protects this organism against antimicrobial effects of aminoglycosides [65]. As eDNA is considered a key component of the *P. aeruginosa* EPS matrix and under intense research, further aspects of its relevance for structure and functions of biofilms can be expected.

## 7. Physical Properties of EPS

Sutherland called the matrix “*a strong and sticky framework*” [66] Cohesion and mechanical stability of EPS components are provided by weak physico-chemical interactions and entanglements of proteins and polysaccharides [48,67], nucleic acids [68] as well as by curli, fimbriae [69], cellulose [70] and amyloids [71]. The role of the individual EPS components can be very important. For example, the biofilm matrix of *P. aeruginosa* becomes more viscous without Psl, which facilitates biofilm spreading [72]. Another example is acetylation of alginate in the same organism: mutants with non-acetylated alginate showed significantly lower cohesiveness [20]. Hydrodynamic conditions contribute to the wide variety of biofilm morphologies. In response to shear stress, the matrix firstly showed properties of an elastic body, which turned into a viscoelastic liquid upon exceeding a given breaking point [73,74]. Under stagnant or low-flow conditions, fewer cohesive biofilms are observed, while thinner and stronger biofilms emerge under high-flow conditions [75]. The biofilm can respond by formation of ripples and even rolling along a surface with increasing shear stress [76], a phenomenon, which has been explained by the quorum-sensing controlled secretion of biosurfactants [77]. Shear stress also influences the composition, dynamics and diversity of biofilms [78], suggesting that high shear stress decreases biofilm diversity and the analysis of biofilm community dynamics. They assume that shear stress would slow down biofilm maturation and tend to maintain a “young” biofilm. Using a load-compression tester and a combination of wild type bacteria, mutants and chemical treatments, Peterson et al. [79] showed that eDNA played a distinct role in the visco-elastic properties of biofilms. The relaxation properties attributed to eDNA could clearly be separated from the roles of the polysaccharides and these studies demonstrate that eDNA plays an important role in mediating the cohesiveness of the biofilm. This is reflected in studies where biofilms that lack eDNA are more easily removed when exposed to surfactant stress [80].

The fact that the structure is non-rigid allows for movement of biofilm organisms in the matrix with consequences for porosity, mechanical properties and microrheology [79,81]. Not all cells in the matrix are immobilized but some of them can move. Common observations include the vertical migration of bacterial populations, e.g., in hypersaline microbial mats [82]. Migration has been observed to be a collaborative effort which includes division of labour [83]. They reported that “*Bacillus subtilis* migrates over a solid surface by forming multicellular structures. In this case, migration depends on the synergistic interaction of two cell types: surfactin-producing and matrix-producing cells. Surfactin-producing cells facilitate migration by reducing the friction between cells and their substrate, thereby allowing matrix-producing cells to organize themselves into bundles that form filamentous loops at the colony edge”. A clear collective behaviour is swarming, which, however, is propelled by flagellae [84]. Surface-active substances contribute to the emergent properties of biofilms. It is well known that rhamnolipids facilitate movement of *P. aeruginosa* in biofilms [85], but they are distributed among diverse microbial genera [86]. Surfactin, formed by *B. subtilis,* inhibits biofilm formation of different strains, including *E. coli*, *P. mirabilis* and *S. enterica* [87]. Sometimes, movement is surprisingly fast. In a spectacular study, Houry et al. [49] discovered that “a subpopulation of planktonic *Bacillus thuringensis* which is propelled by flagellae, was able to tunnel deep within a biofilm structure. “Swimmers” create transient pores that increase mass transfer within the biofilm. Invasion of the biofilm by swimmer bacteria may improve biofilm bacterial fitness by creating channels, increasing nutrient flow into the matrix”. However, the authors depicted mobility also as a competition strategy: “Swimmers can exacerbate killing of biofilm bacteria by facilitating penetration of toxic substances from the environment. They combined these observations with the fact that numerous bacteria produce antimicrobial substances in nature and showed that motile bacilli, expressing a bactericide, can make a heterologous biofilm population over 100 times more sensitive to biocides” (in that study, it was *Staphylococcus aureus*) and then take over the newly created space. It can be concluded that it is not uncommon that biofilm cells actively change their location. Pores and channels between microcolonies that form voids in the matrix [88] were recently shown to facilitate liquid transport [89], inspiring the concept of a “rudimentary circulation system” for the biofilm [90].

Fabbri and Stoodley [91] gave an excellent review in which they demonstrated that it is not only the biology of EPS that contributes to viscoelasticity and structure but also the biophysical factors under which the biofilm is grown. Herewith, mechanical properties are determined by an interaction between a structure’s composition and external environmental changes, which may provide possible survival fitness. Biofilm adaptation to persist on surfaces involves structural and physiological changes, which are represented in the viscoelasticity of a biofilm.

The optical properties of the EPS matrix are also of—little acknowledged—relevance, e.g., for photosynthetic organisms in microbial mats. Decho et al., [92] found that EPS layers have a slightly different refractive index than water. For that reason, they “forward scatter” photons instead of reflecting them by backscattering. Consequently, light shows a tendency to enter the biofilm instead of being than reflected from the surface. A similar process (forward scattering due to change in refractive index) can be observed in a white sandy beach (most of photons are “backscattered” to observer, hence the bright white colour). When incoming waves are “wetting” the sand, they put a layer of water around the grains. This, in turn, results in slight changes of the index of refraction, and photons are “forward scattered” (away from the observer). This is the reason why the “wet sand looks darker”, an equivalent to the “wet T-shirt effect”. However, due to gender objections, the phenomenon was eventually termed as “biofilm gel effect”. Finally, a gel-like EPS spreads out the sand grains, allowing more light to enter into the sand. The EPS gel allows the “recapture” of reflected or scattered photons from an underlying surface. Thus, an increase the chances of absorption is observed due to cellular chromophores [92]. This effect certainly provides light for photosynthetic organisms at the bottom of biofilms. From such observations, it seems certainly possible that the EPS matrix can function as a light conductor. Unfortunately, this path of research has not been pursued further.

## 8. Self-Organization of the Matrix

**Figure 5:**
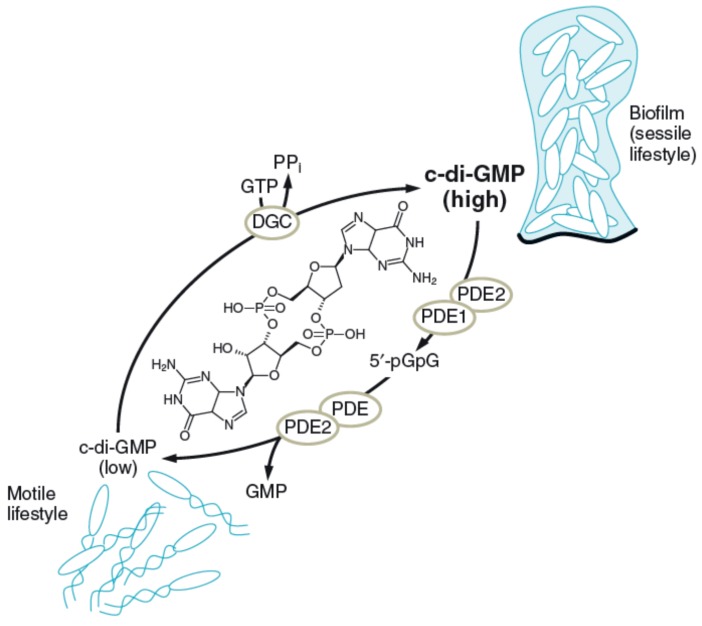
Control of biofilm formation by c-di-Guanidinemonophosphate (c-di-GMP). Concentration of c-di-GMP is regulated by DGC (Diguanylate cyclase; GGDEF domain: diguanylate cyclase), PDE 1 (Phosphodiesterase; EAL domain: diguanylate phosphodiesterase, linearizes c-di-GMP to 5′-pGpG), PDE (nonspecific cellular PDEs, further degrading 5′-pGpG to GMP), and PDE 2 (Phosphodiesterase; HD-GYP domain, metal dependent, unrelated to the EAL domain, linearizes c-di-GMP to 5′-pGpG, degrades 5′-pGpG further to GMP). pGpG—5′ phosphoguanylyl(3′,5′)guanosine; GMP—Guanosine-5-phosphate (from Krauss, G.-J., Nies, D. (Eds.): Ecological Biochemistry-Environmental and Interspecies Interactions today, VCH Weinheim, 2014, with permission).

Self-organization within the matrix occurs based on the activity of the organisms present. An interesting example of how biofilm organisms practice self-organization is offered by *Bacillus subtilis.* When growth slows due to nutrient depletion, nearly all bacteria are encased in the matrix, which is triggered by nutrient depletion [93], and the mature biofilm looks macroscopically wrinkled. In the late stages of *B. subtilis* biofilm development, severe starvation occurs and hundreds of cells pile up in aerial structures known as fruiting bodies that serve as preferential sites for sporulation [89]. An example for more complex regulation of adhesion and detachment is the role of cyclic di-Guanosinemonophosphate (c-di-GMP) in a range of microbial species [94,95]: as a secondary messenger, it stimulates the biosynthesis of adhesins and EPS matrix substances in biofilms. Furthermore, it inhibits various forms of motility and thus, it essentially controls the transition between the motile planktonic and sessile biofilm-associated modes of life of bacteria. The synthesis and degradation of c-di-GMP can be triggered by environmental signals, controlling the c-di-GMP concentration in the cell. At high concentrations, the cell joins a biofilm; at low concentrations, it prefers the planktonic style of life (Figure 5).

In very elegant studies investigating *E. coli*, Serra et al. [36,69] found “clear spatial physiological differentiation, complex supracellular architecture and striking morphology in macrocolony biofilms, controlled by the cyclic-di-GMP levels in the cells. Gradients of nutrients, oxygen, waste products and signalling molecules determine whether bacteria grow and proliferate or enter into stationary phase, using their remaining resources for maintenance and survival. Particularly interesting is the role of flagella as an architectural element. In E. coli macrocolonies, the bottom zone featured elongated dividing cells and a tight mesh of entangled flagellae, which was only possible with active flagellar motor function. The cells in the outer-rim zone produce flagellae, which wrap around and tether the cells together. Adjacent to this growth zone, small chains and patches of shorter curli-surrounded cells appear side by side with flagellated curli-free cells before curly finally becomes confluent hith essentially all cells in the surface layer being encased in ‘curli baskets’”.

## 9. Matrix Dispersal—An Organized Process

The matrix is clearly one of the defining features of the biofilm and is largely responsible for the cohesion of cells within the biofilm. At the end of the biofilm life-cycle, bacteria can transit back to the planktonic phase, through the processes of passive, e.g., sloughing or erosion, and active, dispersal, processes. Dispersal has been shown to be genetically controlled in response to a range of signals and cues such that bacteria can control the switch from a sessile to a free life-style. Given that dispersal has been widely reviewed (e.g., [96,97]), instead the focus here will be put on those processes integral to the matrix. Effectively, active dispersal requires bacteria to break out of their self-made prison (or home) formed from various proteins, polysaccharides and extra-cellular DNA etc. Some bacteria, such as *Pseudomoans putida* utilise large, amyloid like proteins to anchor themselves to surfaces. Under starvation conditions, the intracellular concentrations of c-di-GMP change in *P. putida*, resulting in a change in activity of the protease LapG. It is this protease that ultimately then cleaves the LapA protein to release the cells from the substratum, effectively cutting the anchors so that the cells can swim away [97,98]. Similarly, some bacteria encode polysaccharide degrading enzymes, such as alginate lyase, chitinase or dispersin, that are induced during dispersal to breakdown these matrix polymers [99]. There is also clear evidence that bacteria encode nucleases to degrade the third of the major matrix biomolecules, eDNA [100].

**Figure 6:**
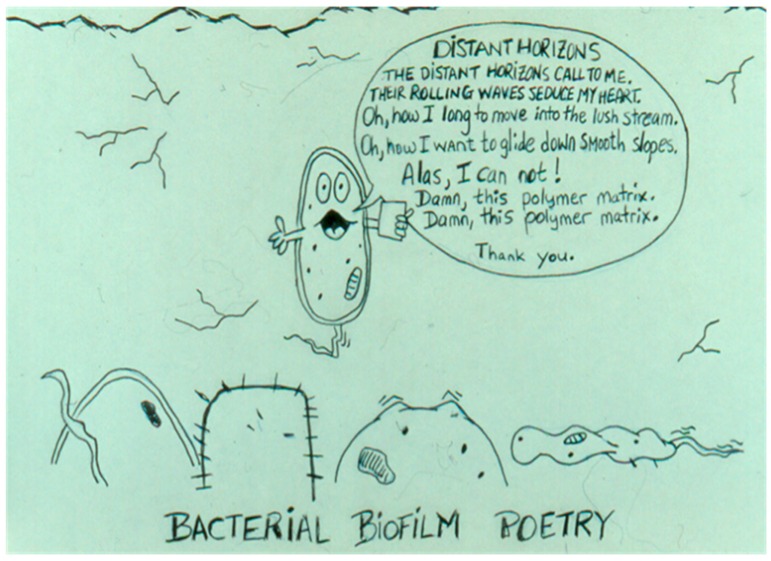
The dammned polymer matrix (J.C. Bryers, Univ. Washington, with permission).

These examples only scratch the surface of the vast array of specific matrix components and it seems likely that enzymes to break these bonds are encoded by the relevant organisms. However, it is also possible that the activity of such enzymes is not limited to dispersal alone, but may also be important in the remodelling of the biofilm matrix as it matures or adapts to changing environmental conditions, such as hydrodynamic shear. In support of this, changes in the microrheological properties of *P. aeruginosa* biofilms change over the developmental cycle [79] and whether this is solely due to changes in the production of specific polysaccharides or is intimately tied to the activity of polysaccharide degrading enzymes remains to be discovered. Obviously, there are mechanisms for biofilm organisms to leave the matrix—it is not their prison although it may have its restrictions, as illustrated in Figure 6.

## 10. Good Enough to Be True

It took a long time until the EPS matrix began to gain the attention, which was appropriate to its relevance. In fact, life may have well originated on wet surface in prebiotic gels, undergoing a transition to a living biofilm, as suggested by Trevors [101]. This is not so far from Haeckel’s theory. From then on, they became an unparalleled success model in evolution. The oldest references for life on Earth are stromatolites, dating back 3.5–3.7 billion years [102,103], and they were generated by biofilms, growing on top of each other. Their construction material was EPS. They must have been involved in the evolution of life, and, thus, involved in the process of “anabiosis”, as postulated by Haeckel, but in a slightly different sense. In addition, if there is life as we know it, i.e., carbon-based and water-dependent, elsewhere in the universe, the best chances are that its representatives are microorganisms—and probably, they live on surfaces as biofilms, organized in an EPS matrix.

It seems as if “slime”, an unsightly everyday phenomenon, has been very much under-appreciated for long. In the meantime it has become obvious that the EPS matrix is considerably more than just the glue for biofilms. Rather, it is a highly sophisticated functional scaffold, which grants the biofilm mode of life its particular and successful features.

Inevitably, all this opened a Pandora’s box full of more questions. Some of them (called “challenges”) are compiled here (after [104,105]):

- What is the distance over which the microbial cell is able to control its external microhabitat?
- What is the nature of the matrix in relation to the genotype, phenotype and environmental cues? How is the dynamic of matrix expression controlled?
- What influences EPS production and how can it be managed?
- Which are the interactions of the various EPS components?
- Which components contribute to matrix stability and how can we influence them?
- Where and by which mechanisms are hydrophobic substances sorbed and retained in the matrix?
- What are the mechanisms of water retention?
- What is the role of EPS in light transmission to deeper layers of the biofilm?
- What is the fate of released polymers in terms of lifetime, turnover and recycling?

to be continued…

Obviously, there is still much more to explore about the magic of slime!

## Figures and Tables

**Table 1:** Functions of extracellular polymeric substances (EPS) components (after [19], with permission).

Function of EPS Component	Relevance for Biofilm Organism	EPS Components Involved
Adhesion	Initial steps in colonization of abiotic and biotic surfaces by planktonic cells, long-term attachment of whole biofilms to surfaces	Polysaccharides, proteins (e.g., fimbriae), eDNA
Aggregation of bacterial cells	Bridging between cells, (temporary) immobilization of bacterial populations, basis for development of high cell densities, cell-cell recognition	Polysaccharides, proteins, DNA
Cohesion of biofilms	Structural elements forming a hydrated polymer network (biofilm matrix), mediation of mechanical stability of biofilms (frequently in conjunction with multivalent cations or hydrophobic interactions), determination of EPS structure (capsule, slime, sheath) and biofilm architecture, generation of matrix	Neutral and charged polysaccharides, proteins (e.g., amyloids, lectins), DNA
Retention of water	Maintenance of highly hydrated microenvironment around biofilm organisms, dessication tolerance in water-deficient environments	Hydrophilic polysaccharides and Proteins; skin-forming hydrophobic proteins (BslA [46])
Protective barrier against antimicrobials	Resistance to nonspecific and specific host defenses during infection, tolerance to various antimicrobial agents (e.g., disinfectants, antibiotics), protection of cyanobacterial nitrogenase from harmful effects of oxygen; protection against some (but not all!) grazers	Polysaccharides, proteins
Sorption of polar organic compounds	Accumulation of nutrients from the environment, sorption of xenobiotics (detoxification)	Charged or hydrophobic polysaccharides and proteins
Sorption of inorganic ions	Promotion of polysaccharide gel formation, ion exchange, mineral formation, accumulation of toxic metal ions (detoxification)	Charged polysaccharides and proteins, including inorganic substituents such as phosphate and sulphate
Sorption of apolar organic substances	Resource capture	Proteins, not yet defined hydrophobic pockets in matrix
Sorption of particles	Resource capture	Sticky matrix components
Enhanced access to resources captured in the matrix	Providing additional enzymatic competence and capacity in the matrix; killer-vesicles as a weapon in competition (Schooling and Beveridge [47]	Membrane vesicles (contain nucleic acids, enzymes, proteins, LPS etc.)
Enzymatic activity	Digestion of exogenous macromolecules for nutrient acquisition, degradation of structural EPS allowing release of cells from biofilms, utilization of substratum as substrate	Proteins
Nutrient source	Source of C, N and P compounds for utilization by biofilm community	Potentially all EPS components
Genetic information	Horizontal gene transfer between biofilm cells	DNA
Intercellular information	Regulation of biofilm dynamics and responses, regulating c-di-GMP concentration	Polysaccharides
		
Electron donor or acceptor	Redox activity in biofilm matrix, electron transport mediation to surfaces	Proteins (e.g., pili, nanowires), humic substances
Resource capture by export of enzymes into matrix	Providing additional enzymatic competence and capacity in the matrix	Outer membrane vesicles (contain nucleic acids, enzyme proteins, lipopolysaccharides, phospholipids)
Sink for excess energy	Sink for excess carbon under unbalanced C:N metabolic conditions	Polysaccharides
Binding of enzymes	Accumulation, retention and stabilization of enzymes through their interaction with polysaccharides	Polysaccharides, enzyme proteins

**Table 2:** Overview of modification of EPS and matrix structure after excretion (after [48], with permission.

Microbial Modification	Effect on EPS
Degradation of EPS components by hydrolases, esterases, lipases, proteases and other lytic enzymes	Shortening of chain length, degradation of EPS, change of matrix structure and stability, formation of pores and channels Destabilization of matrix, dispersion, release of biofilm organisms
Variation of EPS composition in mixed biofilms during development	EPS of different properties, resistance to EPS-lysing enzymes
Post-excretional addition of substituents to polysaccharides	Influence on shape, charge, hydrophobicity of polymer, surface activity
Molecular structure suitable for protein-polysaccharide interaction	Retention, possible protection and activation of extracellular enzymes
Excretion of rhamnolipids	Increase of porosity, favouring of cell motility, influencing mass transport
Movement of “stealth swimmers” Houry et al. [49]	Formation of channels, improvement of convective mass transport
Environmental influence	Effect
Shear forces	Washout of well soluble EPS, accumulation of less soluble EPS, increase of stability of remaining matrix, sloughing off, erosion
Grazing by higher organisms (protozoa, larvae, snails etc.)	Selective removal of EPS and EPS producing organisms, formation of channels, destabilization of matrix

**Table 3:** Cell numbers in biofilms.

System	Cell Density	Reference
*Nitrosomas europeae*	10^10^ mL^−1^	[51]
Activated sludge	10^10^–10^11^ mL^−1^	[52]
Anaerobic granules	~10^11^ g^−1^	Calculated from [53]
Epilithic biofilms	10^8^–10^10^ mL^−1^	[54,55]
Marine snow	10^9^–10^10^ g^−1^	[56]
